# Biomass Waste Inspired Highly Porous Carbon for High Performance Lithium/Sulfur Batteries

**DOI:** 10.3390/nano7090260

**Published:** 2017-09-06

**Authors:** Yan Zhao, Jun Ren, Taizhe Tan, Moulay-Rachid Babaa, Zhumabay Bakenov, Ning Liu, Yongguang Zhang

**Affiliations:** 1 School of Materials Science & Engineering, Research Institute for Energy Equipment Materials, Tianjin Key Laboratory of Materials Laminating Fabrication and Interface Control Technology, Hebei University of Technology, Tianjin 300130, China; yanzhao1984@hebut.edu.cn (Y.Z.); 201631804046@stu.hebut.edu.cn (J.R.); 2 Synergy Innovation Institute of GDUT, Heyuan 517000, Guangdong, China; taizhetan@gdut.edu.cn; 3 Institute of Batteries LLC, 53 Kabanbay Batyr Avenue, Astana 010000, Kazakhstan; mbabaa@nu.edu.kz (M.-R.B.); zbakenov@nu.edu.kz (Z.B.); 4 PI National Laboratory Astana, School of Engineering, Nazarbayev University, 53 Kabanbay Batyr Avenue, Astana 010000, Kazakhstan

**Keywords:** lithium/sulfur battery, cathode, sulfur/highly porous carbon composite, poplar catkin

## Abstract

The synthesis of highly porous carbon (HPC) materials from poplar catkin by KOH chemical activation and hydrothermal carbonization as a conductive additive to a lithium-sulfur cathode is reported. Elemental sulfur was composited with as-prepared HPC through a melt diffusion method to form a S/HPC nanocomposite. Structure and morphology characterization revealed a hierarchically sponge-like structure of HPC with high pore volume (0.62 cm^3^∙g^−1^) and large specific surface area (1261.7 m^2^∙g^−1^). When tested in Li/S batteries, the resulting compound demonstrated excellent cycling stability, delivering a second-specific capacity of 1154 mAh∙g^−1^ as well as presenting 74% retention of value after 100 cycles at 0.1 C. Therefore, the porous structure of HPC plays an important role in enhancing electrochemical properties, which provides conditions for effective charge transfer and effective trapping of soluble polysulfide intermediates, and remarkably improves the electrochemical performance of S/HPC composite cathodes.

## 1. Introduction

As the foremost power source, lithium ion batteries (LIBs) are widely utilized in portable electronic devices such as cell phones and laptops [1]. However, the energy density of current LIBs still lies below that which is demanded by rapidly developing small- and large-scale applications, and especially the requirements of electric vehicles [2]. Recently, lithium/sulfur (Li/S) batteries have received widespread attention because they have considerable specific energy density (2600 Wh·kg^−1^) and large theoretical specific capacity (1675 mAh∙g^−1^). Along with this, elemental sulfur is inexpensive, widely available, ecologically benign and nontoxic [3,4]. However, the application and development of Li/S batteries has been hindered due to two disadvantages; namely, the poor electrochemical conductivity and accessibility of sulfur, as well as the high solubility of lithium polysulfides in organic liquid electrolyte in the electrochemical reaction process, resulting in low electrochemical reversibility and rapid capacity loss upon operation [5,6].

Various strategic approaches have been employed to address these challenges, including using solid or gel polymer electrolytes and the incorporation of sulfur into different conductive matrices [7,8,9,10,11,12,13,14,15]. Among these methods, compositing sulfur with porous carbon materials, which act as a polysulfide absorber and electrically conductive matrix for charge transfer, was proven to be an effective way to enhance Li/S battery performance [10,11,12,13]. Porous carbon materials possess a large specific surface area for efficient sulfur uptake, and high pore volume can provide enough space to accommodate large volume change. The interconnected pore structure can not only improve conductivity and dispersion of S, but also restrict polysulfide diffusion within the frameworks. The excellent electrical conductivity of porous carbon materials can improve the electronic insulation of sulfur and its reduction products. However, synthesis of porous carbon materials for these purposes usually involves complex multistep processes, and uses exotic templates or surfactants, involves complex process control, and uses high-cost carbon sources, which restricts their widespread application [14]. To overcome these obstacles, exploration of renewable biomass as starting materials to synthesize functional carbon materials would be absolutely worthwhile, especially considering the high carbon content and simplicity and low cost of synthesis of carbonaceous materials from them [15].

In our previous study, poplar catkin, a non-economically valued biowaste, was shown to be an ideal carbon precursor for obtaining carbon microtubes (CMT) through a simple one-step carbonization, which were then further composited with sulfur to prepare S/C composite electrode materials and Li/S batteries [16]. Very recently, Li et al. [17] successfully converted catkins into interconnected porous carbon nanosheets (PCN) by KOH activation and heat treatment. These PCNs were used as electrode materials for supercapacitors, and displayed a high specific energy of 21 Wh·kg^−1^ at 180 W·kg^−1^. Compared with a counterpart material prepared without KOH activation, PCNs possessed a much higher surface area of about 1590 m^2^∙g^−1^.

Inspired by this work, we herein used highly porous carbon (HPC) derived from poplar catkins to composite with sulfur to prepare high-performance cathodes for Li/S batteries, and studied the composition, structure, morphology and electrochemical performance of the S/HPC composite.

## 2. Results and Discussion

**Figure 1:**
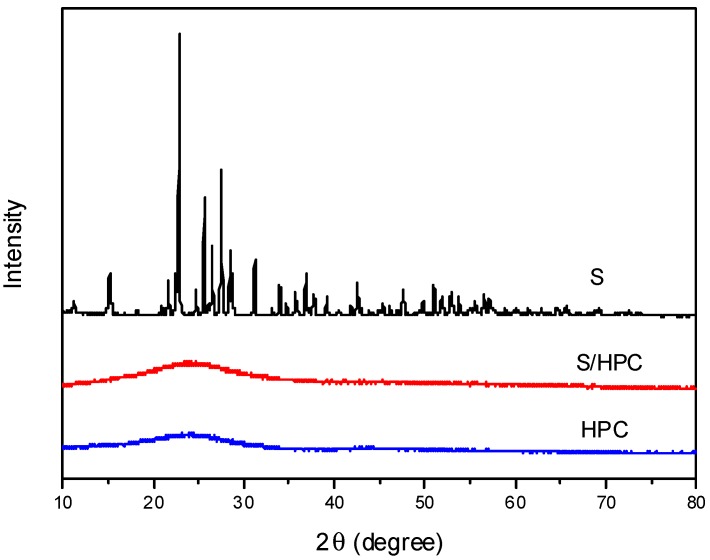
XRD patterns of S, HPC and S/HPC composite.

Figure 1 displays XRD spectra for sulfur, HPC and S/HPC composite. One can see that the as-prepared HPC is amorphous in the XRD spectrum, and the sharp diffraction peaks of S exhibit the features of a crystalline state. The characteristic peaks of elemental sulfur vanish after heat treatment, indicating that, in the composite, the embedded sulfur that was trapped inside the microporous and/or mesoporous structure exists as highly-dispersed fine particles and is unable to crystallize [18].

**Figure 2:**
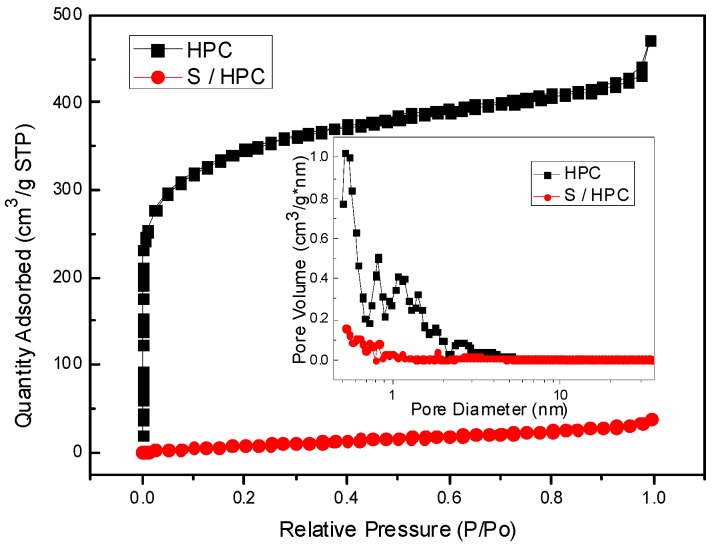
N_2_ adsorption-desorption isotherms and pore size distribution of HPC and S/HPC composite.

Figure 2 shows the Nitrogen sorption isotherms of the HPC of type I without a hysteresis loop with a surface area of about 1261.7 m^2^∙g^−1^ by BET method; the pore size distribution, which ranges from 0.5 to 2.0 nm, is displayed as an inset in Figure 2, with a pore volume of 0.62 cm^3^∙g^−1^, indicating the microporous structure of HPC. However, for the S/HPC composite, the specific surface area has decreased significantly to only 42.1 m^2^∙g^−1^, while the pore volume remained 0.05 cm^3^∙g^−1^, which shows a sharp decline because of the incorporation of most of the sulfur into the interior pore structure of HPC, as was suggested by the XRD results.

**Figure 3:**
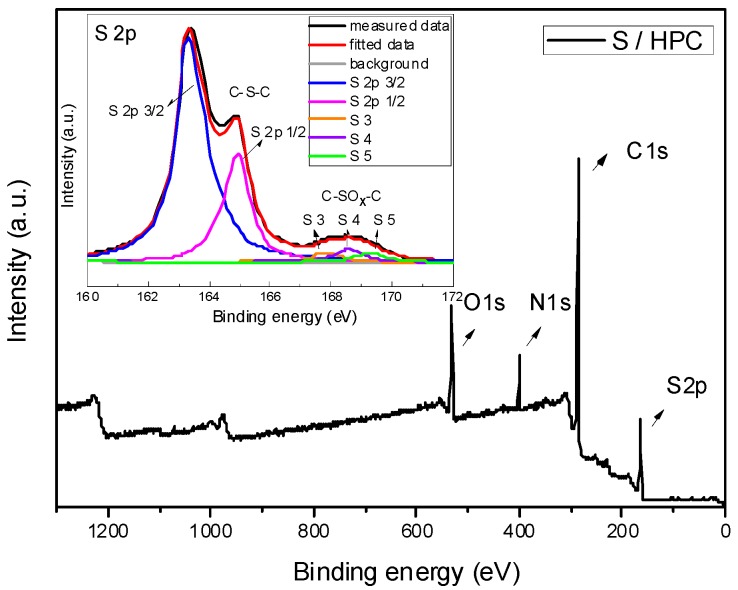
XPS spectra and high-resolution spectra of S 2p of S/HPC composite.

In order to examine the surface chemical properties of the S/HPC composite, XPS measurements were performed. The XPS spectra of the composite are presented in Figure 3. The survey scan spectrum of S/HPC composite shows four peaks, centered at 284.6, 400.5, 531.6 and 166.3 ± 3.3 eV, and corresponding to C 1s, N 1s, O 1s, and S 2p, respectively. Meanwhile, there is no remarkable change in the typical peaks of sulfur (S 2p) after heat treatment. The S 2p spectra of S/HPC composite can be characterized by five peaks at binding energies of 163.6, 164.8, 167.7, 168.6 and 169.3 eV, corresponding to S 2p3/2, S 2p1/2, S3, S4 and S5 groups, respectively. The two prominent peaks correspond to S 2p3/2 and S 2p1/2 of the C-S-C covalent bond of thiophene-S caused by a spin-orbit coupling. The other three broad, low-intensity peaks of S3, S4 and S5 were appointed to different oxidized forms of sulfur, such as C-SO_x_-C (x = 2–4) bonds [19,20,21,22]. These results suggest that, after heat treatment, sulfur is embedded in micropores of the composite with chemical interaction with the carbon material.

**Figure 4:**
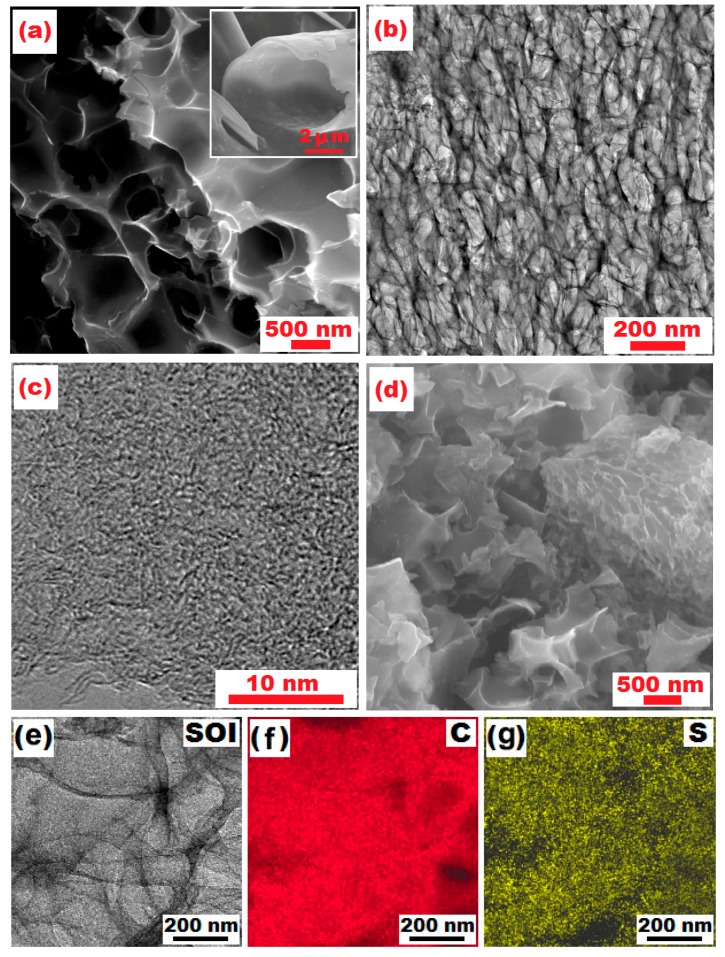
(**a**) SEM image of HPC, inset: SEM image of carbon microtube derived from catkin without KOH activation; (**b**,**c**) TEM images of HPC at different magnifications; (**d**) SEM image of S/HPC composite; (**e**–**g**) TEM image of S/HPC sample and EDS mapping showing distribution of C and S.

The micro-nano structure of the HPC sample was investigated by SEM and TEM. One can clearly see in the inset of Figure 4a that the raw catkin has a three-dimensionally open-ended tubular structure with micrometer-range diameters. After heat treatment and KOH activation, HPC was synthesized, displaying a hierarchically sponge-like structure with nanometer-level pores (Figure 4a). The typical TEM image of HPC shown in Figure 4b, demonstrates a three-dimensionally porous structure of the material, which could incorporate sulfur and suppress diffusion of dissolved polysulfides to electrolyte [23].

From the higher magnification TEM image (Figure 4c), no obvious lattice fringes can be observed, which verifies the amorphous characteristics of prepared HPC, and is in agreement with the XRD results above. The SEM image shown in Figure 4d further demonstrates the S/HPC composite maintains the three-dimensional porous structure observed in the HPC. We find no differences in morphology between the S/HPC binary material and the HPC, which indicates that the sulfur could be evenly distributed within the micropores of the HPC. This porous and loose architecture of the S/HPC composite with a homogeneous distribution of sulfur could facilitate the transport of Li-ions and electrons more efficiently between electrode and electrolyte, greatly favoring the occurrence of the electrochemical reaction of Li/S redox process. The EDS elemental mappings of S/HPC composite was carried out as shown in Figure 4e–g, and the corresponding elemental mappings of C and S demonstrate the existence and homogeneous distribution of S.

**Figure 5:**
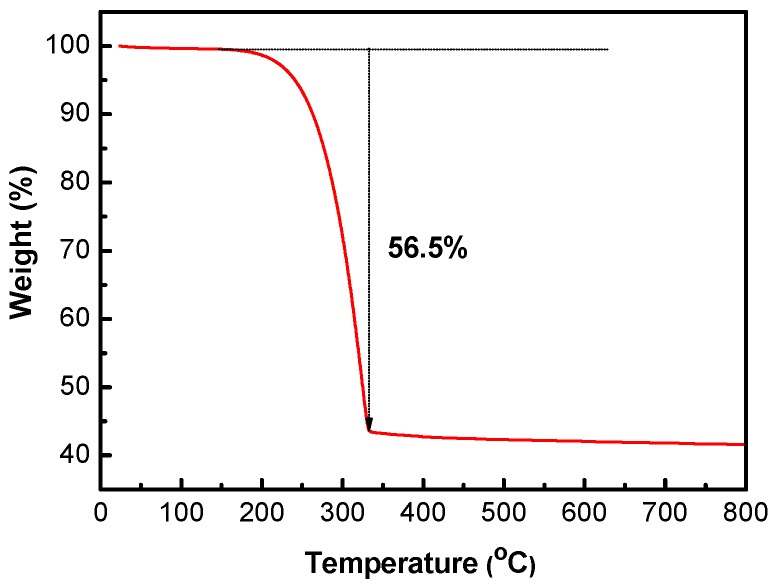
TGA plot of S/HPC composite.

The sulfur content of the S/HPC composite was determined by thermogravimetric analysis (TGA), as shown in Figure 5. The sulfur content of S/HPC was around 56.5%. There is a slight loss of weight below 120 °C, which can be ascribed to the evaporation of adsorbed water molecules. Moreover, the sulfur component in the S/HPC composite totally evaporates at an elevated temperature of 320 °C. High sulfur content in composites will ensure the high overall energy density per gram of cathode in Li/S batteries.

**Figure 6:**
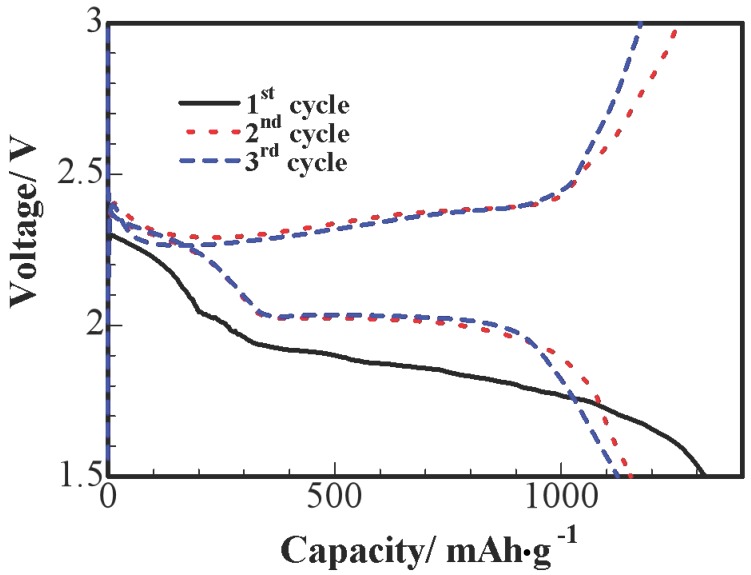
Charge/discharge potential profiles of S/HPC composite cathode at 0.1 C.

Figure 6 shows the three primary discharge and charge voltage curves of S/HPC nanocomposites at 0.1 C; we can observe an obvious reduction process at the initial cycle. This is probably due to an activation process during the initial discharge and the formation of a solid electrolyte interface (SEI), and these processes leading to a larger polarization between the reduction and oxidation peaks. In the 2nd and 3rd cycles, potential profiles show two apparent discharge plateaus, which correspond to a two-step reaction of S→S_n_^2−^(n ≥ 4)→S_2_^2−^. The first short plateau is shown at 2.3–2.4 V vs. Li^+^/Li, due to the formation of long-chain polysulfides (Li_2_Sn, n ≥ 4), which could be dissolved in the liquid electrolyte. The sustained plateau at about 2.0 V reflects the electrochemical transition of the polysulfides to lithium sulfide Li_2_S [10]. It can be seen that no remarkable difference is observed at the 2.0 V discharge plateaus after the initial cycle, the lower-voltage plateau diminishes and almost disappears after the initial cycle.

**Figure 7:**
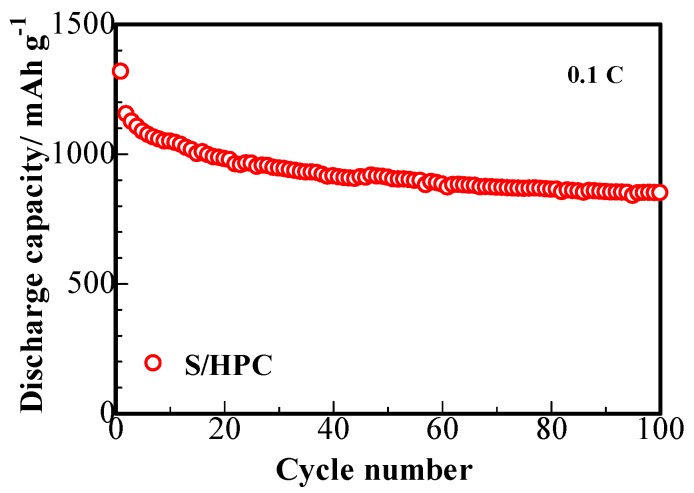
Cycling performance of S/HPC composite cathode at 0.1 C.

Figure 7 presents the cycling capability of S/HPC composites. The cathode displays a large primary discharge capacity of 1318 mAh∙g^−1^ at 0.1 C and the fading trend displays a superior cycling stability of 850 mAh∙g^−1^ reversible capacity after 100 cycles. This phenomenon delivers an increase of at least 40 mAh∙g^−1^ reversible specific capacity, compared to that of the sulfur composite prepared with the carbon microtube derived from catkin without KOH activation [16]. This enhanced electrochemical performance of S/HPC composite cathode with the KOH activated HPC are owing to the hierarchical porous structure of HPC, providing improved absorption ability for the composite to retain the polysulfides dissolved in the electrolyte. As was shown by the SEM, TEM and BET studies, the HPC provides a larger chemical interaction area, which can boost the electrochemical activity of the cathode and maintain higher usage efficiency of active sulfur during the redox reactions [16].

**Figure 8:**
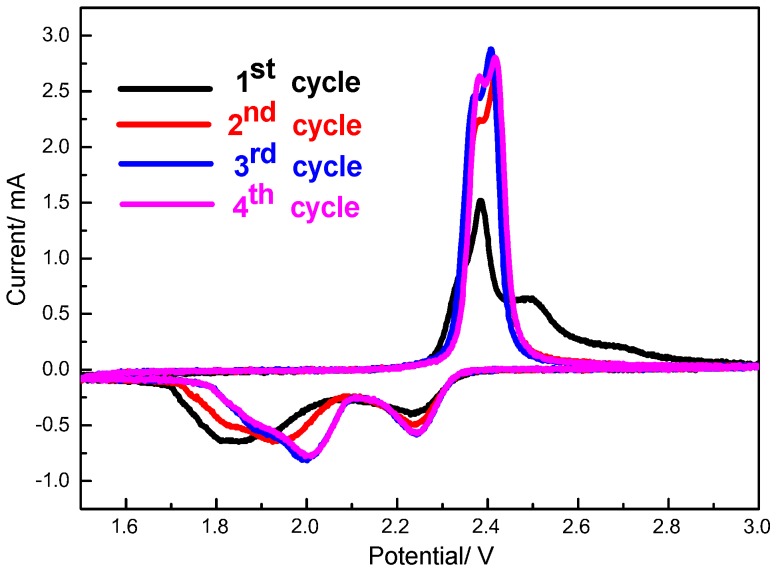
CV profiles of S/HPC composite cathode (the potential sweep rate is 0.1 mV∙s^−1^).

Figure 8 displays the cyclic voltammograms of S/HPC cathode in the initial four cycles. As shown, two cathodic peaks and two anodic peaks are observed from the first four cycles. The two cathodic peaks corresponding to the two-step reduction of sulfur. Two partially overlapping oxidation peaks can be assigned to the reversible conversion of Li_2_S to low-order polysulfides and then to high-order polysulfides, respectively. It can be seen that there is an activation process during the initial discharge, and the cathodic peaks are obviously shifted to higher voltage from the second cycle to the fourth cycle. This reveals that the electrochemical reactions need to overcome the strong energy absorption between S and the conductive matrix [24]. At the following cycles, the S/HPC composite displays that the 3rd and 4th cycle curves nearly overlap with each other. This could be attributed to the excellent reversibility and high stability of the S/HPC composite electrode.

**Figure 9:**
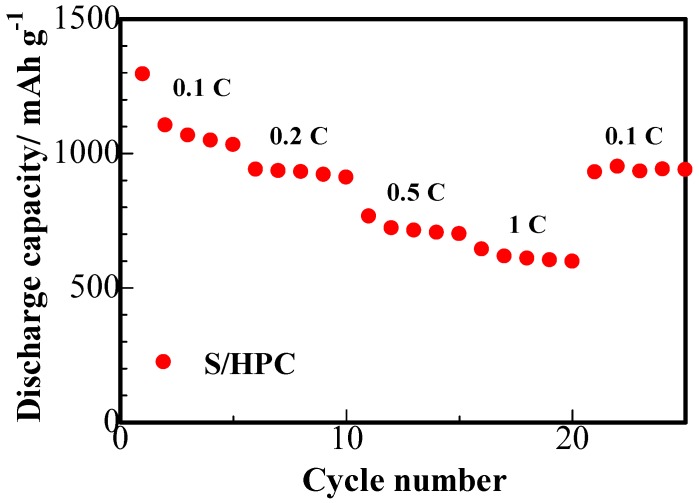
Rate capability of the lithium cells with S/HPC composite cathode.

The results of the rate capability studies of the S/HPC binary cathode are displayed in Figure 9. It can be seen that the S/HPC composite possess a high initial capacity of 1294 mAh·g^−1^ at 0.1 C. With an increase in C rate to 0.2, 0.5 and 1 C, the discharge capacity of the electrode gradually decreases to 934, 705 and 617 mAh·g^−1^, respectively. Furthermore, the discharge capacity was mostly recovered when charge-discharge rate was reduced once more to 0.1 C. Compared to the S/porous carbon composite cathode previously reported in the literature [16,25], the S/HPC composite cathode achieved in our work exhibits a highly reversible capacity and rate capability. This indicates that the S/HPC composite is highly robust and electrochemically stable.

## 3. Materials and Methods

The poplar catkins were collected from a nearby square in Xigu Park, Tianjin, China. In order to develop a facile and scalable synthesis method, catkin was transformed into the porous carbon material by a simple route as follows. Before use, 3 g of catkins were washed by deionized water to remove impurities and dried adequately at 60 °C. Then the sample was stirred for 2 h in KOH aqueous solution (10 mol dm^−3^) with a catkin/KOH ratio of 1:1 by weight, filtered using a glass-fiber filter and dried thoroughly in a dryer at 60 °C. The obtained products were further carbonized at 700 °C for 1 h in argon. The resulting solid sample was treated by HCl (1 mol L^−1^) and then washed with deionized water to pH = 7. Then the residue was dried in a dryer at 90 °C overnight. This highly porous carbon derived from catkin is denoted subsequently as HPC. 0.2 g HPC powder and 4g nano-sulfur suspension (Shanghai Huzheng Nano Technology, 10 wt %) were mixed together by ultrasonic homogenizer (Fisher Scientific, FB120) for 2 h, and vacuum dried at 60 °C for 12 h. Then the S/HPC composite was processed by heating at 150 °C in an Ar-flowing tube furnace and maintained at this temperature for 3 h.

The crystal phase of samples was examined by the X-ray diffractometry (XRD, smart lab, Rigaku Corporation Tokyo, Japan). The sample surfaces were investigated using scanning electron microscopy (SEM, Nova Nano SEM-450, FEI, Eindhoven, The Netherlands) and their interior morphology was characterized by transmission electron microscopy (TEM, JEM-2100F, JEOL, Tokyo, Japan). Chemical analysis (Vario EL, Micro cube, Elementar, Hanau, Germany) was applied to measure the content of S in the S/HPC composite. N_2_ adsorption/desorption measurements were measured by adsorption analyzer (ASAP 2020 M + C, Micromeritics, Atlanta, GA, USA) at 77 K. The pore size distribution curves and specific surface area of the powders were calculated according to the Barrett-Joyner-Halenda (BJH) method and Brunauer-Emmett-Teller (BET) method from adsorption, respectively. X-ray photoelectron spectroscopy (XPS) was used to explore the surface chemical properties of the composite (Thermo Scientific ESCALAB 250Xi, Waltham, MA, USA). The S content in S/HPC composite was measured by thermogravimetric analysis (TGA, STD Q-600, TA Instruments-Waters LLC, Newcastle, DE, USA).

The CR2025 coin cell was assembled to detect the electrochemical performance of S/HPC composite with a glove box full of high purity argon (99.9995% purity). Lithium metal was utilized as a counter electrode, in addition to the S/HPC composite cathode, sandwiching a microporous polypropylene separator between them. The electrolyte consisted of 1 mol dm-3solution of lithium bis (trifluoromethane) sulfonamide (LiTFSI, Aldrich, Darmstadt, Germany, 96% purity) in tetraethylene glycol dimethyl ether (Aldrich, 99% purity). Working electrode slurry was fabricated by blending active material (S/HPC sample), polyvinylidene fluoride (PVDF) and acetylene black (AB) with a weight ratio of 8:1:1 in the *N*-methyl-2-pyrrolidinone (NMP) solvent. Then, the resulting slurry was homogeneously coated onto an aluminum current collector and subsequently evaporated under a vacuum environment at 65 °C for 12 h. The cell tests were performed using a battery tester (Neware Inc., Shenzhen, China) within a potential range of 1.5–3.0 V vs. Li^+^/Li at 25 °C. The CV test was carried out using an electrochemical workstation (Princeton, VersaSTAT 4, 50/60 Hz, Ametek, PA, USA).

## 4. Conclusions

In summary, the successful preparation of a highly porous carbon (HPC) was demonstrated using both pyrolysis and KOH activation of the poplar catkin. The prepared HPC was composited with sulfur via a melt-diffusion route to obtain a high performance composite sulfur cathode. Due to its three-dimensionally porous structure, the S/HPC composite cathode displays a good cycling and rate performance which exhibits a high initial discharge capacity of 1318 mAh∙g^−1^, while maintaining a reversible capacity of 850 mAh∙g^−1^ after 100 cycles at 0.1 C. With an eye to the large number of researchers currently concerned with applications of 3D connected porous carbon structures for Li/S batteries, this study opens new possibilities for a more efficient and low-cost utilization of ‘trapped’ sulfur from waste biomass resources.

## References

[1] Zhang Y., Wei Y., Li H., Zhao Y., Yin F. (2016). Simple fabrication of free-standing ZnO/graphene/carbon nanotube composite anode for lithium-ion batteries. Mater. Lett..

[2] Manthiram A., Fu Y., Chuang S., Zu C., Su Y. (2014). Rechargeable lithium-sulfur batteries. Chem. Rev..

[3] Bruce P., Freunberger S., Hardwick L., Tarascon J. (2012). Li-O_2_ and Li-S batteries with high energy storage. Nat. Mater..

[4] Zhang Y., Zhao Y., Bakenov Z., Tuiyebayeva M., Konarov A., Chen P. (2014). Synthesis of hierarchical porous sulfur/polypyrrole/multiwalled carbon nanotube composite cathode for lithium batteries. Electrochim. Acta.

[5] Konarov A., Gosselink D., Doan T., Zhang Y., Zhao Y., Chen P. (2014). Simple, scalable, and economical preparation of sulfur-PAN composite cathodes for Li/S batteries. J. Power Sources.

[6] Zhao Y., Yin F., Zhang Y., Zhang C., Mentbayeva A., Umirov N., Xie H., Bakenov Z. (2015). A Free-Standing Sulfur/Nitrogen-Doped Carbon Nanotube Electrode for High-Performance Lithium/Sulfur Batteries. Nanoscale Res. Lett..

[7] Tu Z., Nath P., Lu Y., Tikekar M., Archer L. (2015). Nanostructured Electrolytes for Stable Lithium Electrodeposition in Secondary Batteries. Acc. Chem. Res..

[8] Zhang C., Lin Y., Liu J. (2015). Sulfur double locked by a macro-structural cathode and a solid polymer electrolyte for lithium-sulfur batteries. J. Mater. Chem. A.

[9] Zhao Y., Zhang Y., Bakenov Z., Chen P. (2013). Electrochemical performance of lithium gel polymer battery with nanostructured sulfur/carbon composite cathode. Solid State Ion..

[10] Kim J., Kim H., Ahn J., Lee K., Yoo W., Sung Y. (2016). Activation of micropore-confined sulfur within hierarchical porous carbon for lithium-sulfur batteries. J. Power Sources.

[11] Seo S., Choi C., Kim D. (2016). Fabrication of sulfur-impregnated porous carbon nanostructured electrodes via dual-mode activation for lithium-sulfur batteries. Mater. Lett..

[12] Li H., Wang Z., Zhang Y., Wang X., Zhao Y., Maximov M., Ji P., Yin F. (2016). Interconnected nitrogen-doped carbon nanofibers derived from polypyrrole for high-performance Li/S batteries. Russ. J. Appl. Chem..

[13] Chen F., Yang J., Bai T., Long B., Zhou X. (2016). Biomass waste-derived honeycomb-like nitrogen and oxygen dual-doped porous carbon for high performance lithium-sulfur batteries. Electrochim. Acta.

[14] Yin F., Liu X., Zhang Y., Zhao Y., Menbayeva A., Bakenov Z., Wang X. (2017). Well-dispersed sulfur anchored on interconnected polypyrrole nanofiber network as high performance cathode for lithium-sulfur batteries. Solid State Sci..

[15] Wang J., Xie K., Wei B. (2015). Advanced engineering of nanostructured carbons for lithium-sulfur batteries. Nano Energy.

[16] Zhang Y., Zhao Y., Konarov A., Li Z., Chen P. (2015). Effect of mesoporous carbon microtube prepared by carbonizing the poplar catkin on sulfur cathode performance in Li/S batteries. J. Alloys Compd..

[17] Li Y., Wang G., Wei T., Fan Z., Yan P. (2016). Nitrogen and sulfur co-doped porous carbon nanosheets derived from willow catkin for supercapacitors. Nano Energy.

[18] Zhang Y., Zhao Y., Bakenov Z., Babaa M., Konarov A., Ding C., Chen P. (2013). Effect of graphene on sulfur/polyacrylonitrile nanocomposite cathode in high performance lithium/sulfur batteries. J. Electrochem. Soc..

[19] Wang Y., Huang L., Sun L., Xie S., Xu G., Chen S., Xu Y., Li J., Chou S., Dou S. (2012). Facile synthesis of a interleaved expanded graphite-embedded sulphur nanocomposite as cathode of Li-S batteries with excellent lithium storage performance. J. Mater. Chem..

[20] Xin S., Gu L., Zhao N., Tin Y., Zhou L., Guo Y., Wan L. (2012). Smaller sulfur molecules promise better lithium-sulfur batteries. J. Am. Chem. Soc..

[21] Yu X., Park H. (2014). Sulfur-incorporated, porous graphene films for high performance flexible electrochemical capacitors. Carbon.

[22] Yang J., Wang S., Ma Z., Du Z., Li C., Song J., Wang J., Shao J. (2015). Novel nitrogen-doped hierarchically porous coralloid carbon materials as host matrixes for lithium-sulfur batteries. Electrochim. Acta.

[23] Liang C., Dudney N., Howe J. (2009). Hierarchically structured sulfur/carbon nanocomposite material for high-energy lithium battery. Chem. Mater..

[24] Liang X., Zhang M., Kaiser M.R., Gao X., Konstantinov K., Tandiono R., Wang Z., Liu H.K., Dou S.X., Wang J. (2015). Split-half-tubular polypyrrole@sulfur@polypyrrole composite with a novel three-layer-3D structure as cathode for lithium/sulfur batteries. Nano Energy.

[25] Zhu Y., Xu G., Zhang X., Wang S., Li C., Wang G. (2017). Hierarchical porous carbon derived from soybean hulls as a cathode matrix for lithium-sulfur batteries. J. Alloys Compd..

